# A Rare Case of Tinea Capitis in Morocco Due to Trichophyton tonsurans

**DOI:** 10.7759/cureus.63273

**Published:** 2024-06-27

**Authors:** Hanaa Nejjari, Imane Zouaoui, Sara Aoufi

**Affiliations:** 1 Central Laboratory of Parasitology and Mycology, Ibn Sina University Hospital, Faculty of Medicine and Pharmacy, Mohammed V University, Rabat, MAR

**Keywords:** morocco, hygiene, mycology, judo, tinea capitis, tricophyton tonsurans

## Abstract

Tinea capitis is a frequent reason for dermatology consultation in Morocco. In this work, we report a case of Tinea capitis caused by *Trichophyton tonsurans *(*T. tonsurans*), an unusual anthropophilic dermatophyte in Morocco. This pathogen was identified for the first time in our hospital, affecting a young Moroccan judoka. The patient was a 25-year-old man. He was a member of the Moroccan national judo team. He was sent to the parasitology and mycology laboratory for suspicion of tinea capitis. The anamnesis found an almost annual participation in international tournaments and competitions. The clinical examination revealed erythematous-squamous scalp plaque associated with hair loss and two localized squamous lesions on the right wrist and the left knee. We sampled the lesions separately. Direct examination in potassium hydroxide preparation of collected samples (skin scrapings, hair fragments) from the patient's lesions was negative, and cultures grew *T. tonsurans* in Sabouraud Agar. We identified this pathogenic fungal species based on the colonies' macroscopic and microscopic morphological characteristics, establishing the diagnosis of *T. tonsurans *tinea capitis. The young judoka presented an unusual fungal infection of the scalp in Morocco. We suppose it to be our country's first case of *T. tonsurans* tinea capitis. Screening international combat sports practitioners and optimizing hygiene conditions in our sports environments remains necessary to avoid any epidemic of *T. tonsurans*.

## Introduction

Tinea capitis is a fungal infection caused by keratinophilic filamentous fungi (dermatophytes). It is a common reason for dermatology consultations in Morocco, mainly affecting young children [1]. In this study, we report a case of tinea capitis associated with dermatophytic lesions caused by *Trichophyton tonsurans* (T. tonsurans), an unusual anthropophilic dermatophyte in Morocco [2-3]. To our knowledge, this is the first identification of this species in our hospital, affecting a young Moroccan judoka.

## Case presentation

The patient, a 25-year-old Moroccan man with no history of diabetes or medical treatment, had been practicing judo since the age of 6 and was a member of the Moroccan national judo team. He was referred to our parasitology and mycology laboratory for suspected tinea capitis. During the interview, the young judoka reported his almost yearly participation in international tournaments and competitions.

**Figure 1:**
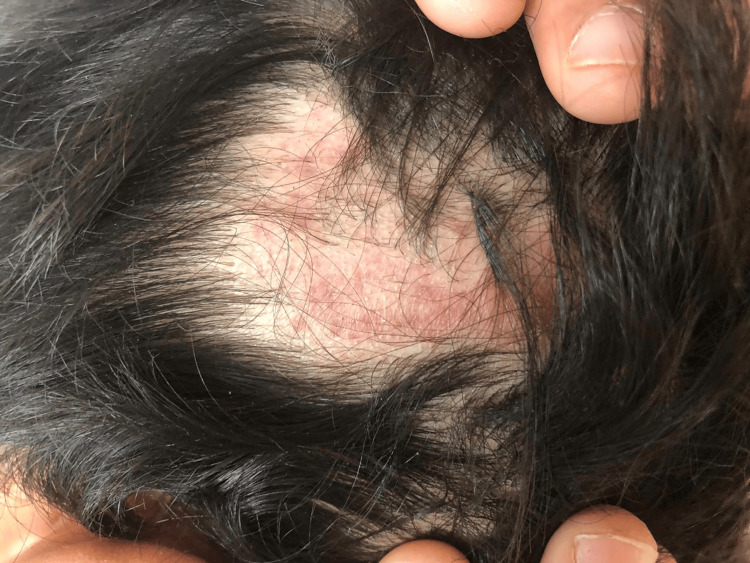
Erythematous-squamous plaque of the patient’s scalp

**Figure 2:**
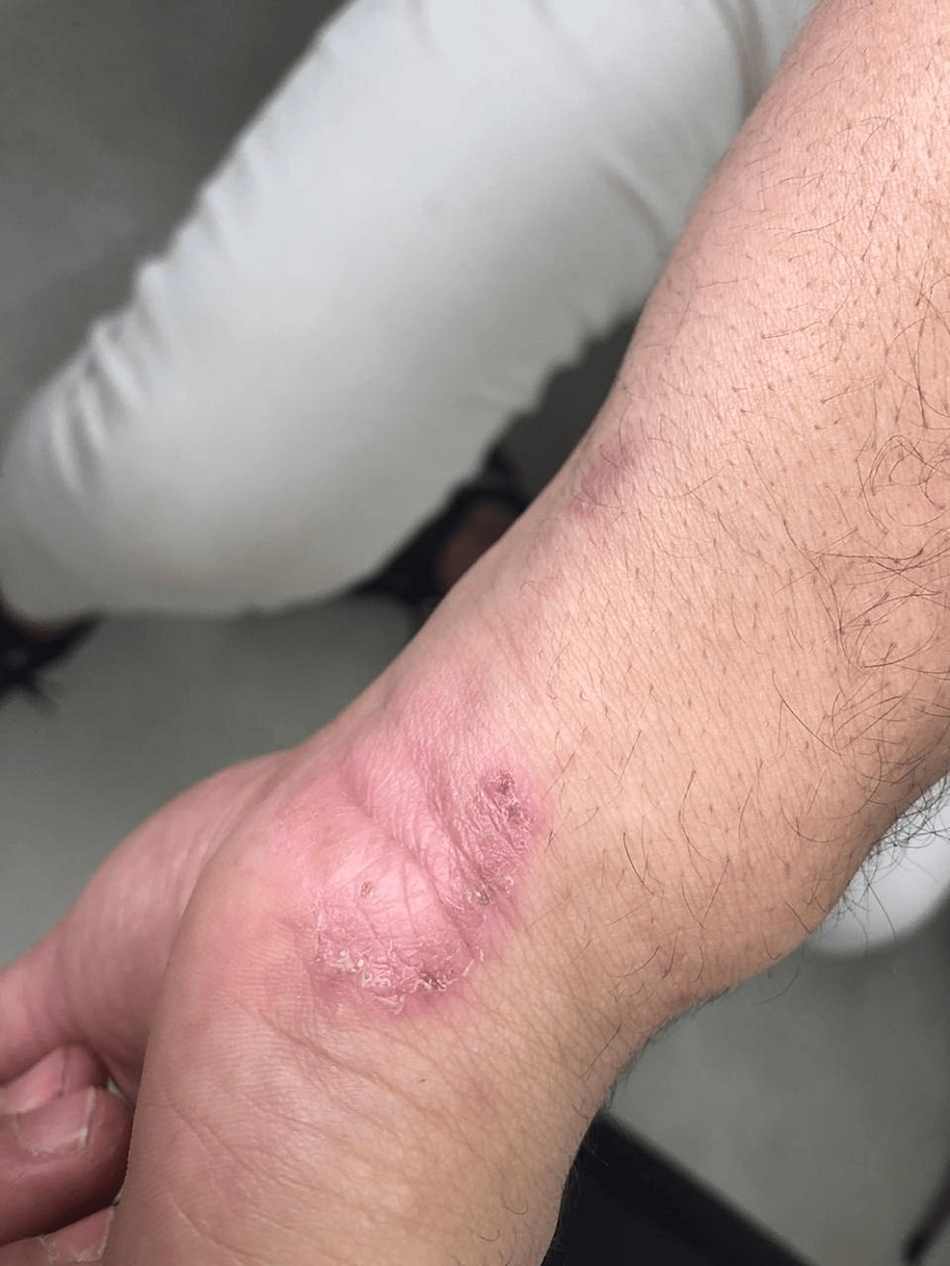
Lesion on the right wrist anterolateral region

**Figure 3:**
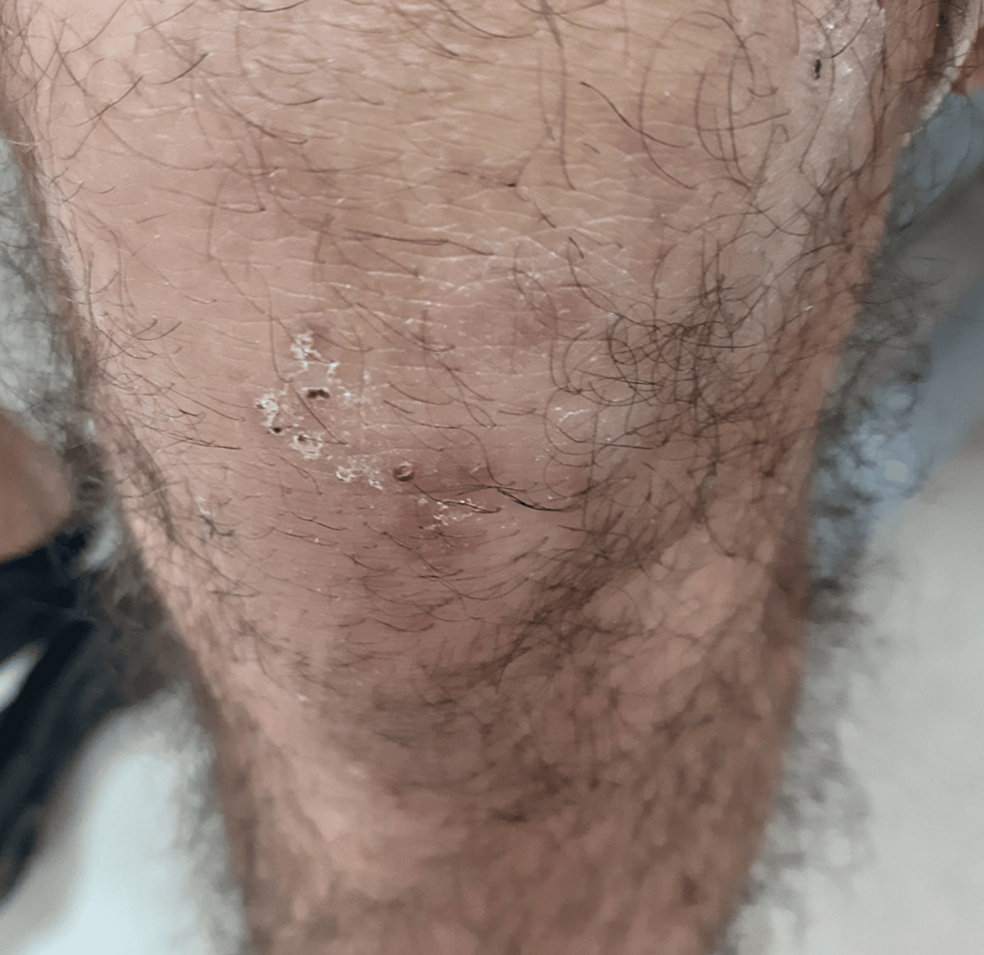
Lesion on the left knee anterior region

Clinical examination revealed a single erythematous-squamous plaque on the scalp, irregular in contour, approximately 7 cm in diameter, nonpruritic, painless, associated with hair loss in this area (Figure 1), and two irregular squamous lesions on the anterolateral aspect of the right wrist (Figure 2) and the anterior aspect of the left knee (Figure 3). We sampled the lesions separately.

A scalpel blade was used to scrape the scales from the junctions between the healthy and diseased areas of the three affected sites, and a few broken hairs were removed from the periphery of the scalp plaque. Biological samples were collected in sterile Petri dishes.

**Figure 4:**
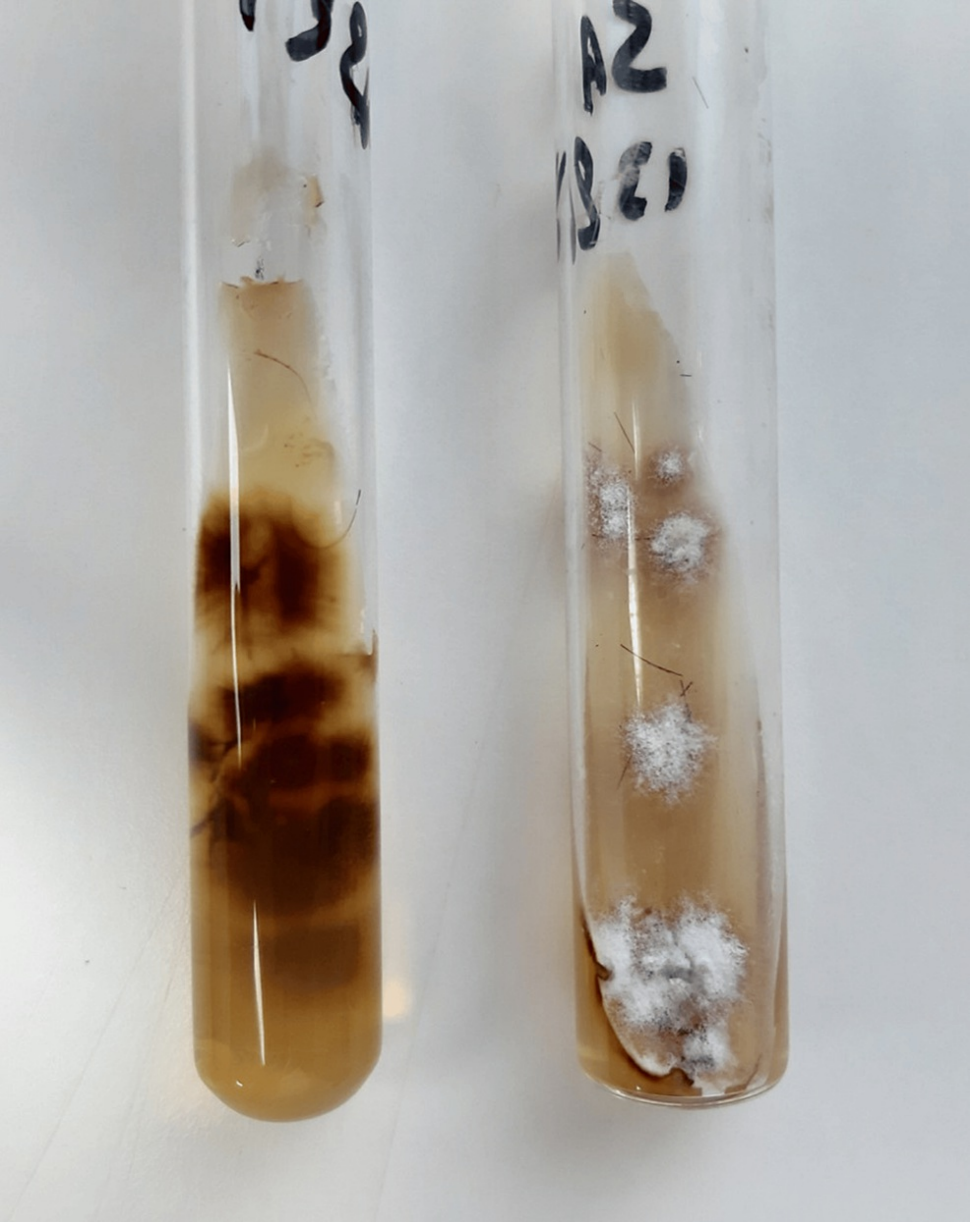
Growth of Trichophyton tonsurans in culture

Direct examination of samples collected from the patient's lesions in a 30% potassium hydroxide preparation was negative. Hairs and scales were cultured in Sabouraud chloramphenicol actidione agar, in which white and powdery colonies with a brown pigmented undersurface grew after 10 days of incubation at 27°C and 37°C (Figure 4).

**Figure 5:**
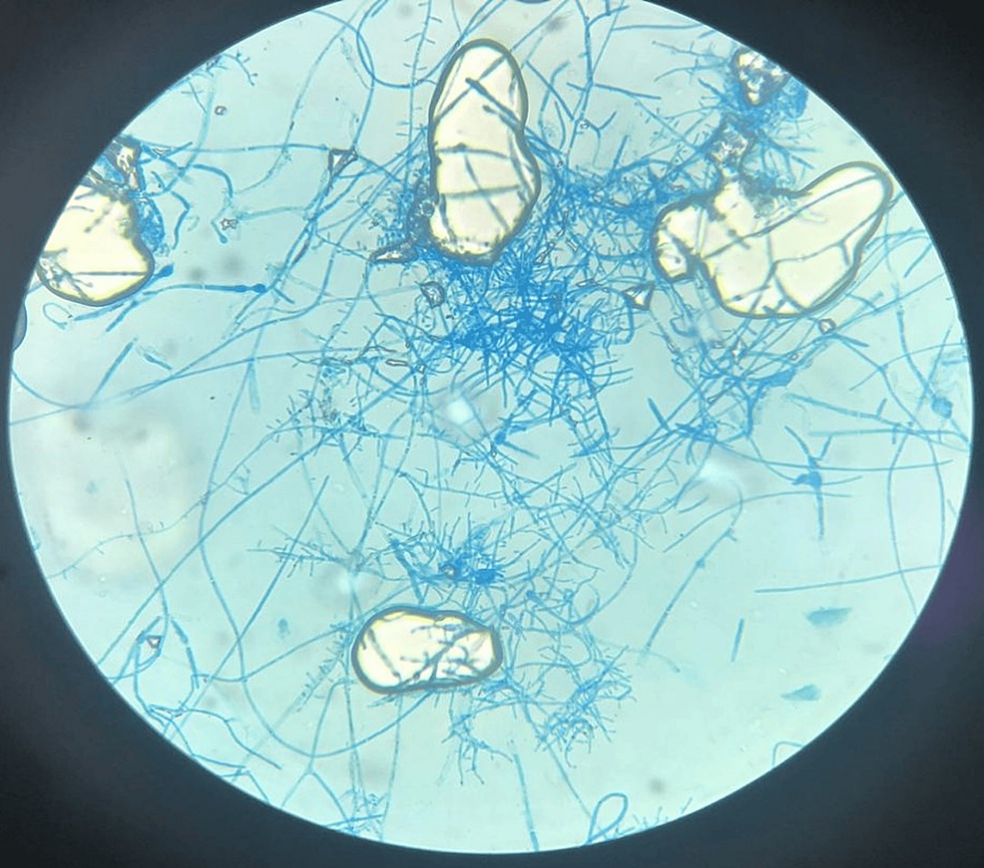
Right-angle branching and pyriform microconidia (40x magnification)

**Figure 6:**
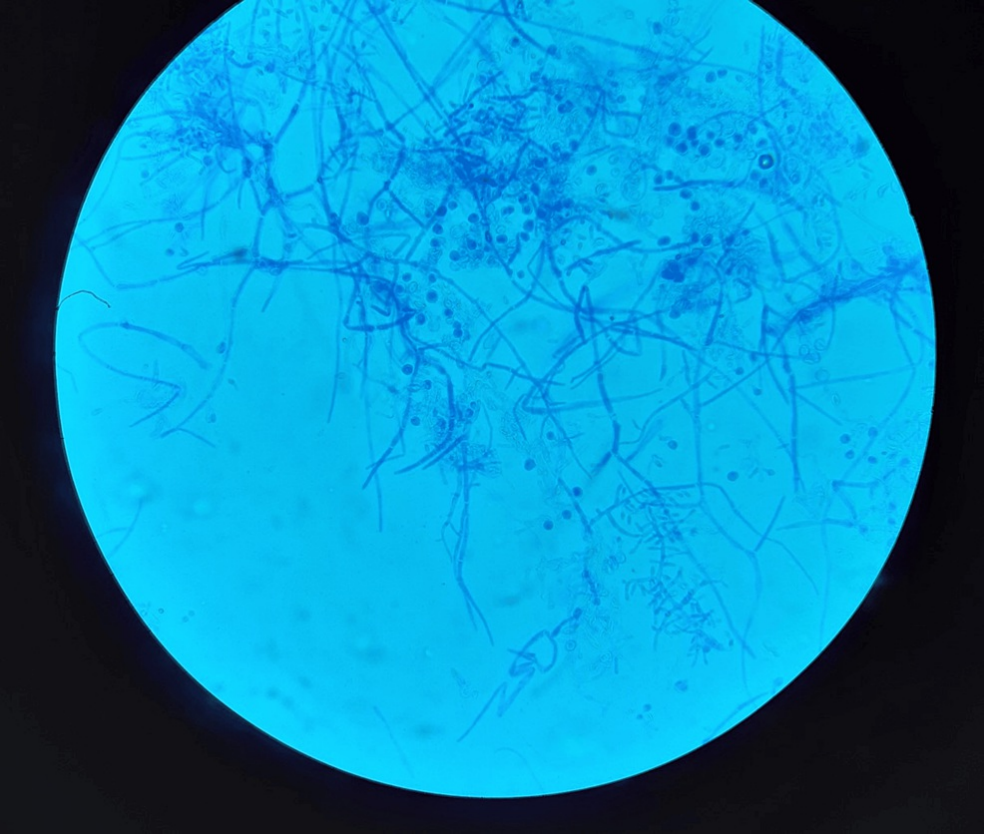
Chlamydospores seen under optical microscope (40x magnification)

Microscopic examination of these colonies in lactophenol blue revealed mycelial filaments with short ramifications perpendicularly projected from hyphae, numerous round or pyriform microconidia with acladium disposition (Figure 5), and terminal or intercalary chlamydospores (Figure 6). 

The species T. tonsurans was identified. The patient was referred to our laboratory as an outpatient. After the diagnosis was made, he was lost to follow up without leaving any contact information. Consequently, we do not know the treatment he received or the progression of his disease.

## Discussion

T. tonsurans is an anthropophilic dermatophyte of the Ascomycota phylum. It is rare in Morocco and globally distributed in the United States, England, Canada, Brazil, France, and Japan [4]. This filamentous fungus is primarily responsible for tinea corporis and tinea capitis, but kerions and onychomycosis may also be observed [2-3,5].

In martial arts, these lesions are likely to be contagious, mainly through prolonged direct contact (skin-to-skin) [6] or indirect contact (Kimonos, mats, hairbrushes, etc.) [7], leading to epidemics in groups practicing this type of sport, including judo. Recently, T. tonsurans dermatophytosis outbreaks in fighting sports teams have been described [8-9].

No case of T. tonsurans tinea capitis has been reported in Morocco. Our patient would be the first observed case. It is probably an importation related to his frequent participation in international judo competitions.

Our literature review allowed us to identify two cases of T. tonsurans reported in Morocco: This dermatophyte was first isolated in 2005 from a patient with onychomycosis of the big toes [2]. The second case published in 2016 involved a patient with onychomycosis of the hands [3]. In the Maghreb region, two cases of T. tonsurans tinea capitis were diagnosed in Mauritania [10] and Algeria [11], respectively, in 2019 and 2020.

Clinical diagnosis of T. tonsurans mycosis is often difficult. In the scalp, the clinical spectrum of this anthropophilic fungus is broad, ranging from a simple seborrhoeic form characterized by pellicles and crusts [8] to inflammatory forms (Kerion) [12]. The classic form is trichophytic tinea capitis with small patches of alopecia, as in our patient.

T. tonsurans causes endothrix hair invasion. This can be detected by directly examining scales and hair mounted with potassium hydroxide preparation. Identification of this dermatophyte is based on the length of growth (approximately 14 days) and the macroscopic and microscopic appearance of the colonies [13].

Medical treatment of T. tonsurans tinea capitis is based on the use of antifungal agents. Topical antifungal agents, such as shampoos or creams containing selenium sulfide, are used to reduce the fungal load on the scalp [14]. However, in order to eradicate the infection completely, it is often necessary to use oral antifungal agents, such as griseofulvin or terbinafine [14]. In addition to medical treatment, it is also necessary to maintain the cleanliness of sports facilities by ensuring that floors, carpets, kimonos, and hairdressing equipment are cleaned and disinfected.

## Conclusions

Diagnosing T. tonsurans dermatophytosis in combat athletes requires screening the patient's contacts to look for an epidemic. To prevent the spread of T. tonsurans in our sports environments and to prevent any epidemic outbreaks, it is crucial to ensure that all individuals involved in combat sports are aware of the importance of maintaining optimal daily hygiene practices (including cleaning and disinfecting the environment), avoiding sharing of personal items, and using individualized headgear and sportswear. Closer collaboration between health professionals and practitioners of international combat sports is required to promote early detection and treatment of injuries.
